# Radiotherapy in Managing Metastatic Hepatocellular Carcinoma With Cardiac Involvement and Pulmonary Tumor Thromboemboli: A Case Report

**DOI:** 10.7759/cureus.36547

**Published:** 2023-03-22

**Authors:** Zeta Chow, Evans Amoah, Zhonglin Hao, Mahesh Kudrimoti

**Affiliations:** 1 Radiation Medicine, University of Kentucky College of Medicine, Lexington, USA; 2 Radiation Oncology, University of Kentucky College of Medicine, Lexington, USA; 3 Medical Oncology, University of Kentucky College of Medicine, Lexington, USA

**Keywords:** palliative radiation, tumor thromboemboli, hepatocellular carcinoma, radiation, radiotherapy

## Abstract

Hepatocellular carcinoma (HCC) is the most common liver cancer and presents various degrees of aggressiveness. In this case study, we reported the management of an aggressive HCC patient who was a young immigrant from a hepatitis B endemic country with locally advanced HCC with portal involvement at presentation. Patient was initially managed with Yttrium-90 (Y-90) instillation, then systemic treatment when he had disease progression. Despite multiple lines of systemic treatments, patient continued to progress and developed significant cardiac involvement and pulmonary tumor thromboemboli. His course of treatment was further complicated by hemoptysis, presumably from hemorrhagic tumor thromboemboli. Patient became ineligible for systemic treatment due to the risk of hemoptysis, thus, subsequently managed with a course of palliative radiotherapy. Unfortunately, patient developed hemorrhagic shock, cardiac failure, and septic shock during radiation treatment and expired shortly afterward. In this case report, we discussed multi-modal treatments, including Y-90, systemic treatment, and radiotherapy, in managing complicated and aggressive HCC. We also reported risk factors, prognostic factors, efficacy of Y-90 instillation and the necessity of a personalized treatment approach. In conclusion, there is no consensus on managing patients with metastatic HCC with cardiac and pulmonary involvement currently. Treatment modalities are often highly personalized and require multi-disciplinary discussion.

## Introduction

Hepatocellular carcinoma (HCC) is the most common liver cancer, with rising incidence in the past decade. The incidence of HCC was 6.5 cases per 100,000, which was four times higher compared to 1.5 cases per 100,000 in 1973 [1]. The HCC can be managed with surgical resection, systemic treatment, and radiotherapy, depending on patient characteristics and stage at presentation. While it is not uncommon to have metastatic HCC to lung, bone and lymph nodes, aggressive HCC with cardiac involvement and pulmonary tumor thromboemboli is relatively rare [2]. Currently, there is no consensus and guideline in managing patients with advanced HCC with cardiac and pulmonary artery involvement. Treatment is highly personalized, clinically challenging, and provider-dependent.

In this case report, we presented a patient with aggressive metastatic HCC with cardiac involvement and pulmonary thromboemboli, managed with systemic treatments, Ytterium-90 (Y-90) instillation and also palliative radiotherapy. The Y-90 TheraSphere@ is a Food and Drug Administration-approved radiopharmaceutical conjugated with resin beads. It is injected under image guidance into branches of hepatic arteries to treat localized liver neoplasm, such as HCC and liver metastasis [3]. The purpose of this case study is to report our institutional experience, review current literature, and discuss efficacy and personalized treatments.

## Case presentation

**Figure 1:**
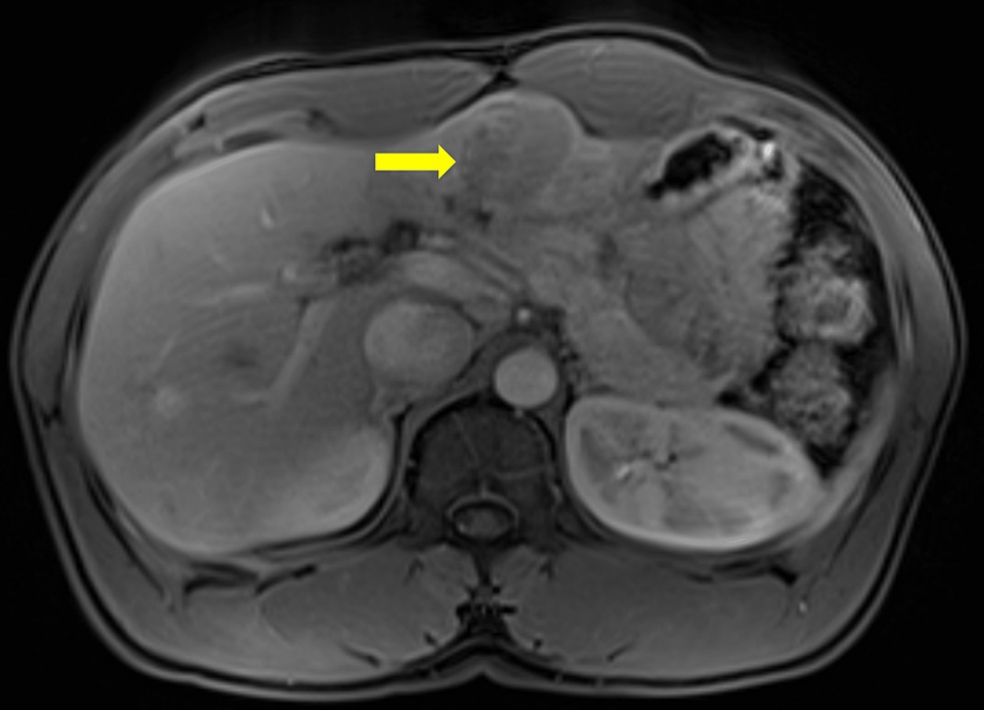
T1-weight axial plane post-contrast venous phase MRI abdomen MRI: Magnetic Resonance Imaging Lesion was measured 4.5 cm and localized to left lateral segments 2 and 3, mildly hypointense with diffuse mild and heterogeneous arterial phase hyperenhancement along with washout and capsule formation.

Patient is a 34-year-old African male immigrant with history of chronic hepatitis B infection who presented with small hepatic lesion on diagnostic ultrasound, while patient was treated for hepatitis B (Tenofovir 300 mg daily). This lesion was biopsied, with pathology consistent with moderately-differentiated hepatocellular carcinoma. On presentation, patient had mild tenderness to palpation due to hepatomegaly without symptoms of hepatic decompensation including jaundice, hepatic encephalopathy, esophageal varices, and ascites. Laboratory work-up revealed elevated Alpha Fetal Protein (AFP) at 3,679.0 ng/mL (normal <10.0 ng/mL), normal Carcinoembryonic Antigen and Carbohydrate Antigen 19-9, and unremarkable renal and liver functions on comprehensive metabolic panel. The Magnetic Resonance Imaging Abdomen with Intravenous (IV) contrast showed a 4.5 cm segment II/III lesion invading the portal and left hepatic vein (Figure 1). Patient was staged as T4N0M0, stage IIIB localized HCC based on American Joint Committee on Cancer staging 8th edition. Multidisciplinary Tumor Board discussion recommended patient to undergo Y-90 instillation for localized HCC.

Interventional radiology successfully performed a mapping angiogram prior to proceeding with Y-90 instillation, which demonstrated extensive enhancement within the left hepatic lobe and tumor thrombus enhancement within portal vein, without significant pulmonary shunting (defined as > 20%). Shortly afterwards, the patient safely received TheraSphere@ Y-90 instillation to the left hepatic lobe according to the mapping angiogram. Based on the volume and location of the tumor, a total of 240 Gray (Gy) to a volume of 550 mL was planned for delivery. Patient had a 5.1% pulmonary shunt and an estimated 1% residual activity at the end of the procedure. Patient tolerated the procedure well and was discharged home. During his 2-week post-procedure follow-up, patient did not have significant acute toxicities and symptomatically improved. The AFP was further elevated to 16,109 ng/mL, with mild transaminitis: Aspartate Aminotransferase 126 U/L (normal 12-40 U/L), Alanine Transaminase 66 U/L (normal 11-41 U/L) and elevation of Alkaline Phosphatase 126 U/L (normal 40-115 U/L). Total bilirubin was 0.3 mg/dL within normal limits (0.2-1.1 mg/mL). Renal function showed normal serum creatinine and estimated glomerular filtrate rate.

**Figure 2:**
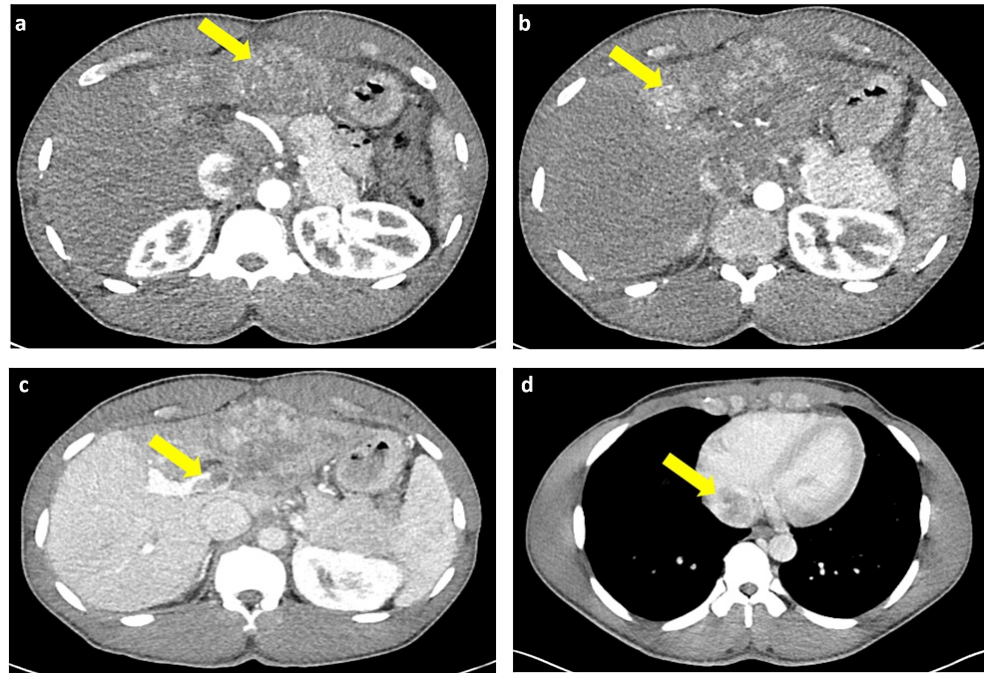
CT abdomen images CT: Computed Tomography a) CT abdomen arterial phase showed a heterogeneously enhancing partially exophytic hepatic lesion arising from the anterior left hepatic lobe, measuring 6 x 5.4cm; b) new heterogeneous enhancing lesion extends into segment 4, measuring 3cm; c) CT abdomen venous phase demonstrated increased enhancing tumor involving the left portal vein with complete obstruction; d) tumor extends into the main right portal vein, present in the left hepatic vein extending into inferior vena cava and right atrium.

Patient underwent Computed Tomography (CT) scans per National Comprehensive Cancer Network guidelines to assess treatment response and to continue disease surveillance at 3-month follow-up. At the time, patient presented with worsening tenderness to palpation of the right upper quadrant and middle abdomen secondary to hepatomegaly. The CT Abdomen and Pelvis with intravenous (IV) contrast scan revealed heterogeneously enhancing partially exophytic hepatic lesion involving segments II, III and IV, complete occlusion of left portal vein and large left hepatic thrombus extending into Inferior Vena Cava and right atrium, suggesting significant progression of disease (Figures 2a-2d). CT Chest with IV contrast revealed small bilateral indeterminate lung nodules without definitive evidence of distal metastasis. His AFP level, though persistently elevated, declined to 3,160 ng/mL and stable hepatic and renal functions. Patient remained functionally well with an Eastern Cooperative Oncology Group score of 0 and Child-Pugh class A.

**Figure 3:**
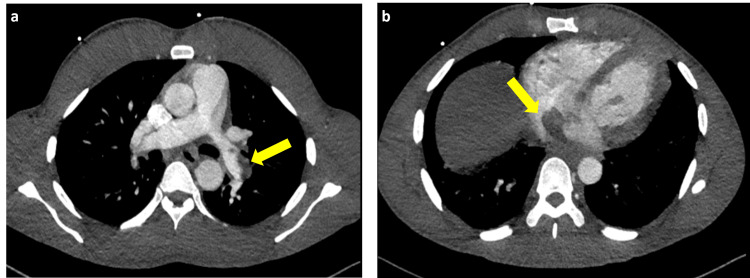
CT Angiogram CT: Computed Tomography; PE: Pulmonary Embolism a) CT Angiogram PE protocol showed partially occlusive thromboemboli within the most distal bilateral main pulmonary arteries extending into upper lobe segmental and subsegmental braches; left main bronchus with the partial filling defect; b) Re-demonstration of filling defect within suprahepatic inferior vena cava with partial protrusion into the right atrium.

**Figure 4:**
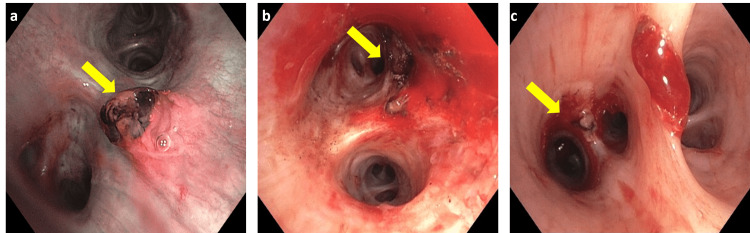
Bronchoscopy images a) Bronchoscopy image of left carina erythematous protruding lesion, without evidence of obstruction. b) post Argon beam ablation of left lung lesion; c) post Argon beam ablation of left upper lobe lesion with evidence of bleeding distal to the lesion.

After an extensive discussion with patient and his family, it was decided that the patient should proceed with systemic therapy with the objective of achieving a treatment response, to make patient a surgical candidate, although unlikely due to the extent and aggressiveness of disease. He then received atezolizumab (1200mg) and bevacizumab (15 mg/kg) every 21 days per IMbrave-150 clinical trial [4]. Patient then started to experience multiple symptoms, including fatigue, weakness, lower extremity pitting edema, and shortness of breath. After receiving two cycles of atezolizumab and bevacizumab, patient developed hemoptysis and presented to the emergency room (ER) for evaluation, during which he was found to have numerous pulmonary metastases, a right-to-left intrapulmonary shunt, and pulmonary thromboemboli on CT Angiogram Pulmonary Emboli protocol (CT-PE) (Figures 3a-3b). Though, his AFP at the time further declined to 2,666 ng/mL. Patient was admitted to the medical intensive care unit for hemoptysis. Bronchoscopy showed dilated ectatic vessel at the carina between left upper lobe and left lower lobe, suspecting main bronchus invasion from pulmonary tumor thromboemboli metastasis (Figures 4a-4c). The lesion was cauterized with Argon ablation and subsequently achieved satisfactory hemostasis. Patient was discharged home afterwards.

During his post-hospitalization visit, atezolizumab and bevacizumab were discontinued due to hemoptysis with concern of increased risk of hemorrhage. In addition, atezoliaumab alone is not known to be better than sorafenib alone. Patient then switched to receiving lenvatinib 12 mg orally daily to continue his systemic treatment. His AFP level stabilized and continued to decline while receiving systemic treatments. Patient condition improved clinically and he was able to return to work briefly.

Unfortunately, after three months of systemic treatment, patient again developed massive hemoptysis and acute respiratory failure, requiring intubation. Repeat CT-PE revealed bilateral pulmonary thromboemboli, portal vein and suprahepatic inferior vena cava involvement and extensive pulmonary metastasis. Patient received serial bronchoscopy while intubated, which did not identify an overt source of bleeding, though observed active bleeding distal to previously identified and cauterized lesion in the left main bronchus, beyond the visualization capacity of bronchoscopy. His bleeding subsequently subsided and he was extubated successfully. Patient was eventually discharged home, but returned in short intervals due to ongoing hemoptysis. His clinical presentation and imaging findings suggested that the pulmonary tumor thromboemboli have eroded into the left bronchi, causing persistent hemoptysis.

After extensive discussion with patient and his family, a course of palliative radiation to the left main bronchial segment and adjacent pulmonary artery with tumor thromboemboli was recommended. The goal of radiation was to temporize bleeding for symptom control, and to potentially restart systemic therapy after achieving hemostasis. Patient was simulated in a supine position with permanent ink marking for alignment purposes. The planned radiation dose was 36 Gy in 24 fractions, 1.5 Gy per fraction, twice a day. Radiation was delivered via Anterior-to-Posterior/Posterior-to-Anterior approach 3-Dimension conformal technique via 6x energy photon beam treatment. 

Initially, the patient responded to radiation treatment and hemoptysis improved during the first week of treatment. However, patient developed significant abdominal and lower extremity swelling, evidence of heart failure due to fluid retention while on treatment. He was medically managed with diuretics. During the second week of radiation treatment, patient presented with massive hemoptysis, hypotension, and tachycardia during on-treatment visit. The patient was emergently transferred to the ER for hemorrhagic shock secondary to massive hemoptysis. He had multiple blood transfusions and his course of hospitalization was further complicated by hepatorenal syndrome, bacterial peritonitis, and sepsis. The patient and his family decided to pursue comfort care measures after the goals of care discussion. Patient expired shortly afterwards, 10 months after his initial diagnosis.

## Discussion

Hepatocellular carcinoma is the most common primary liver cancer. Risk factors for HCC include smoking, alcohol use, non-alcoholic steato-hepatitis and viral hepatitis infections [5]. In the United States, the incidence of liver and intrahepatic bile duct cancers is estimated to reach 41,260 cases and 30,520 deaths in 2022 with greater than 75% attributed to HCC [6]. Among HCC patients, it was reported that about 15% of patients is found to have extrahepatic metastasis at initial presentation [2]. The treatment for HCC is multi-modal, which includes systemic therapy, surgical resection, and radiotherapy.

The main risk factor contributing to the development of HCC in our patient is chronic hepatitis B infection. This is prevalent in African countries, where hepatitis B vaccination and treatment are not readily available to citizens. According to the World Health Organization, the prevalence of hepatitis B infection was 6.1% in Africa, which is higher compared to 0.7 % in North and South America combined [7]. In addition, the prevalence of HCC was 8% in African countries, which is also higher compared to 5% in North America [8]. These developing countries have limited resources, which makes prevention, diagnosis, and treatment of HCC more challenging.

HCC can present as an extremely aggressive disease, as detailed in this case report. Increasing tumor diameter is an important, but not the only prognostic factor for tumor aggressiveness. The AFP increase and percentage of portal vein thrombosis can change exponentially, instead of linearly, as tumor diameter increases [9]. This implies that the biological characteristics may have changed with ultra-high serum AFP and evidence of portal vein thrombosis, predicting a much more aggressive course [9]. Our patient has significantly elevated serum AFP and image evidence of portal vein thrombosis at the presentation; both were poor prognostic factors. Patient presented with a stage II localized HCC and progressed to stage IV metastatic HCC with cardiac and pulmonary involvement in six months, deteriorated quickly and expired in the following six months after multiple lines of treatment. Our patient presented with a significantly more aggressive disease, comparing to the data from the LEGACY study, which reported median progression-free survival and overall survival of 40.7 and 57.9 months respectively for HCC < 8cm undergoing Y-90 instillation [10].

Our patient was treated with both systemic therapy and radiotherapy, including the innovative Y-90 intravascular TheraSphere@ injection. In the PREMIERE trial, a landmark phase 2 study which randomized 45 patients with Barcelona Clinic Liver Cancer early and intermediate stage HCC to undergo conventional Trans-arterial Chemoembolization (cTACE) or Y-90 TheraSphere, Y-90 TheraSphere was shown to significantly improve median time to progression compared to cTACE (>26 months vs 6.8 months, p = 0.0012) [11]. The Y-90 has also been found to have the highest rate of pathologic complete response compared to other locoregional therapies including Radiofrequency Ablation, cTACE and Stereotactic Body Radiotherapy in the setting of bridging HCC patients to transplant [12]. Several clinical trials have assessed the role of Y-90 in combination with systemic treatment, particularly sorafenib, for advanced HCC. The addition of sorafenib to Y-90 was noted to result in increased gastrointestinal and dermatologic toxicities [13]. In regards to patients with liver metastases, multiple studies, especially among metastatic colorectal cancer patients, suggest benefits in local control when Y-90 TheraSphere@ is used alone or in combination with chemotherapy [14]. The use of Y-90 in advanced HCC is recommended based on multidisciplinary tumor board discussion of each individual. Our patient had a centralized segment IV/V lesion abutting the portal vein, which made surgical resection challenging. Y-90 technique was deemed particularly useful given the presence of a centrally located lesion, abutting critical vascular structures in a poor surgical candidate [15].

The most common extrahepatic sites of metastasis for HCC include lungs (47%), lymph nodes (45%) and bones (37%) [2]. Metastatic HCC could present with cardiac or pulmonary artery involvement, but relatively uncommon in literature. There are several case reports regarding HCC with cardiac involvement. Patients often presented with cardiac symptoms including dyspnea on exertion, lower extremity edema and orthopnea [16-18]. Imaging workup showed a mass in either the atrium or ventricle, often right-sided and laboratory markers showed elevated liver enzymes and an abnormal coagulation profile [16-18]. A clinicopathologic study revealed 18 out of 439 cases of patients had HCC with cardiac involvement on autopsy [19]. More recently, it has been estimated that 5-10% of HCC patients may develop cardiac involvement [20]. Despite the uncommon presentation, the prevalence of metastasis to pulmonary artery and cardiac involvement could be higher than expected.

Currently, there is no consensus or study on managing patients with metastatic pulmonary tumor thromboemboli and cardiac involvement. The role of radiotherapy in managing tumor thromboemboli is unclear and has not been established as the standard of care. In this case report, our patient received palliative external beam radiation to the left main bronchus and adjacent pulmonary artery in managing tumor thromboemboli. The radiation regimen was also tailored to his specific clinical scenario, instead of the standard and commonly utilized radiation dose, such as 30 Gy in 10 fractions or 37.5 Gy in 15 fractions. The 36 Gy in 24 fractions radiation dose was used to balance the need for hemostasis and tumor regression. It was thought that a drastic decrease in tumor size in the pulmonary artery might worsen hemoptysis and lead to hemorrhage. The determined treatment was highly personalized and clinician-dependent in this case scenario. There is a need to establish consensus for HCC with cardiac metastasis and tumor thromboembolism in guiding future practice.

## Conclusions

Hepatocellular carcinoma with cardiac involvement and pulmonary thromboemboli is a rare and aggressive presentation and poses a significant challenge in the clinical management of patients. Current treatment options include surgical resection, liver transplant, systemic treatments, external beam radiation, and Y-90 instillation. Management of such an aggressive disease often require multi-disciplinary discussion. In our experience, we offered a multimodal management with a personalized approach in this scenario, considering the patient's functional status and goals of care. Future study is warranted to establish guideline and consensus in managing these patients.
